# Development of search strategies for systematic reviews in health using ChatGPT: a critical analysis

**DOI:** 10.1186/s12967-023-04371-5

**Published:** 2024-01-02

**Authors:** Nathalia Sernizon Guimarães, Julliane Vasconcelos Joviano-Santos, Marcela Gomes Reis, Roberta Rayra Martins Chaves, Observatory of Epidemiology, Nutrition, Health Research (OPENS)

**Affiliations:** Postgraduate Health Science, Medical Sciences College of Minas Gerais, Alameda Ezequiel Dias, 275, Belo Horizonte, Minas Gerais 30130-110 Brazil

## Dear editor

To guide clinical decision-making systematic reviews, need to present transparent, reproducible, and standardized methods for identifying, synthesizing, and describing all scientific literature based on the previously developed central question. After structuring the research question, the strategies for searching for the information are in elaborate sequence. However, it is observed that one of the biggest challenges in designing systematic reviews and meta-analyses in the face of the ascending body of scientific literature is how to be assertive in searching for all scientific information.

ChatGPT is a type of chatbot, developed by free artificial intelligence (AI) model that uses the Generative Pre-trained Transformer language model capable of translating formal and extremely technical information into a clear and simple text in a few minutes [1]. This AI has been trained to access an enormity of data, texts, newspapers and scientific articles been evaluated for validity and execution in scientific research [2–4]. Wang and collaborators [5] tested Boolean operators in 2023, but the structuring a search strategy in different databases has not been critically evaluated yet. In this letter, we present the critical evaluation of ChatGPT’s ability regarding decoding core questions to search the entire literature in three used around the world databases used to guide researchers and methodologists.

To perform the analysis comparative search strategy, we used the record available by the PROSPERO platform (#CRD42023391396) aiming to answer the central question: “When does weight regain occur in obese individuals after bariatric surgery?” The PICOT components of the question were: population—Obese individuals with age major than 18 years; intervention—bariatric surgery; comparator—diet, drugs or placebo; outcomes—time of weight regains and study type—trials.

After structuring the central question, we asked ChatGPT to create the search strategy for the MEDLINE database (Additional file 1: Fig S1) and, after this guidance, we requested the development of search strategies that reflected the central question adapted for two other databases widely used in systematic reviews, LILACS and Embase, with the specific inclusion of descriptor bases (MeSH, DeCS, and Emtree) (Additional file 1: Fig S2). We present the manual search performed by a methodologist and validated by the librarian (Additional file 1: Fig S3).

Despite the quick return, we observed as to the constitution of the search strategies created by ChatGPT that this AI does not insert the synonymous terms (Entry Terms) and the jargon used in the clinical practice of the researchers. As for the structuring, we observed that the search strategies created by ChatGPT do not organize, in a correct manner, the groups of acronyms in the same search key. For example, obese people could not be related as an alternative to weight regain. We also observed that the ChatGPT inserted additional points to the objective of the intervention that was structured by the central question, that is, bariatric surgery and other surgeries are not objects of the central question. The insertion of the search deadline was another important point observed since if not justified, we cannot insert it. At last, we observed that ChatGPT did not insert a validated filter for the limitation of randomized clinical trials. The problems evaluated and the guidelines to work around them are available in Table 1.

**Table 1:** Problems and guidance about the methodological errors observed in the search by ChatGPT

Problems	Guidances
Domain: Contents Lack of terms synonymous with the main search term	**MEDLINE:** it is recommended to insert the main term from the descriptor base (for example: “Bariatric Surgery” [Mesh]) and Entry Terms (for example: “Metabolic Surgery” OR “Metabolic Surgeries” OR “Surgeries, Metabolic” OR “Surgery, Metabolic” OR “Bariatric Surgical Procedures” OR “Bariatric Surgical Procedure” OR “Procedure, Bariatric Surgical” OR “Procedures, Bariatric Surgical” OR “Surgical Procedure, Bariatric” OR “Surgical Procedures, Bariatric” OR “Bariatric Surgeries” OR “Stomach Stapling” OR “Stapling, Stomach”) **Embase.com:** it is recommended to enter the command ‘/syn’
Domain: Content Lack of clinical jargon	It is recommended to use clinical jargon (recognized technical terms not yet indexed by descriptor databases—MeSH. DeCS and Emtree) in search strategies to broaden the evaluation. Example: the term “weight gain” is not indexed in the descriptor databases and is essential for this search strategy because it is the main outcome of the central question
Domain: Structuring Groups of different acronyms in the same search key	The search strategy should be operationalized in Boolean descriptors considering separate keys for the acronym. Example: obese people could not be listed as an alternative to weight regain
Domain: Content Additional keywords to the goal of the intervention that was structured by the central question	The search strategy should be operationalized in Boolean descriptors considering separate keys for the acronym. Example: other surgeries with the exception of bariatric surgery are not the object of the central question
Domain: Content inserting a search deadline	It is recommended that the review be comprehensive, with no time or space restrictions if not justified. Example: In COVID-19, we have a time cutoff of 2020 onwards. However, if we want to research viruses that have caused pandemics, we cannot restrict them
Domain: Content Lack of validated filter	Strategies are designed to retrieve the studies most likely to meet our methodological criteria, such as the type of study that answers the central question. It is recommended, to filter randomized clinical trials in MEDLINE, to use PubMed Special Queries with the following filter: ((clinical[Title/Abstract] AND trial[Title/Abstract]) OR clinical trials as a topic[MeSH Terms] OR clinical trial[Publication Type] OR random*[Title/Abstract] OR random allocation[MeSH Terms] OR therapeutic use[MeSH Subheading])

In conclusion, we recommend caution for conducting information search strategies using ChatGPT exclusively. Despite being a simple-to-run tool and having ease in response, content and structuring problems are reported and searchers should be aware of these problems.

### Supplementary Information

**Additional file 1: Fig S1.** General guidance on building search strategies. **Fig S2.** Specific orientation for the construction of search strategies for information in electronic databases: MEDLINE, Embase.com, and LILACS. **Fig S3.** Manual Search Strategies.

## Data Availability

All data generated or analysed during this study are included in this published article and its supplementary information files. This study utilized data available on public websites and electronic databases. The Embase platform was accessed through the Brazilian government (CAPES website).

## Abbreviations

AI: Artificial intelligence
DeCS: Descritores em Ciências da Saúde
Emtree: Elsevier´s authoritative live science thesaurus
LILACS: Literatura Latino-Americana e do Caribe em Ciências da Saúde
MEDLINE: Medical literature analysis and retrieval system online
MeSH: Medical subject headings
