# Supplementary material for: Development of search strategies for systematic reviews in health using ChatGPT: a critical analysis
Source: J Transl Med. 2024 Jan 2;22:1. doi: 10.1186/s12967-023-04371-5 (PMC10759630; doi:10.1186/s12967-023-04371-5)
Supplement: Supplementary file 1 — Additional file 1: Fig S1. General guidance on building search strategies. Fig S2. Specific orientation for the construction of search strategies for information in electronic databases: MEDLINE, Embase.com, and LILACS. Fig S3. Manual Search Strategies. [file 12967_2023_4371_MOESM1_ESM.docx]

**Additional file – Figures**

**Figure 1** - General guidance on building search strategies.

**Figure 2 -** Specific orientation for the construction of search strategies for information in electronic databases: MEDLINE, Embase.com, and LILACS.

**Figure 3** - Manual Search Strategies.


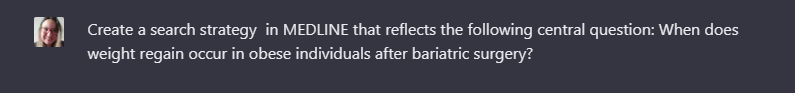

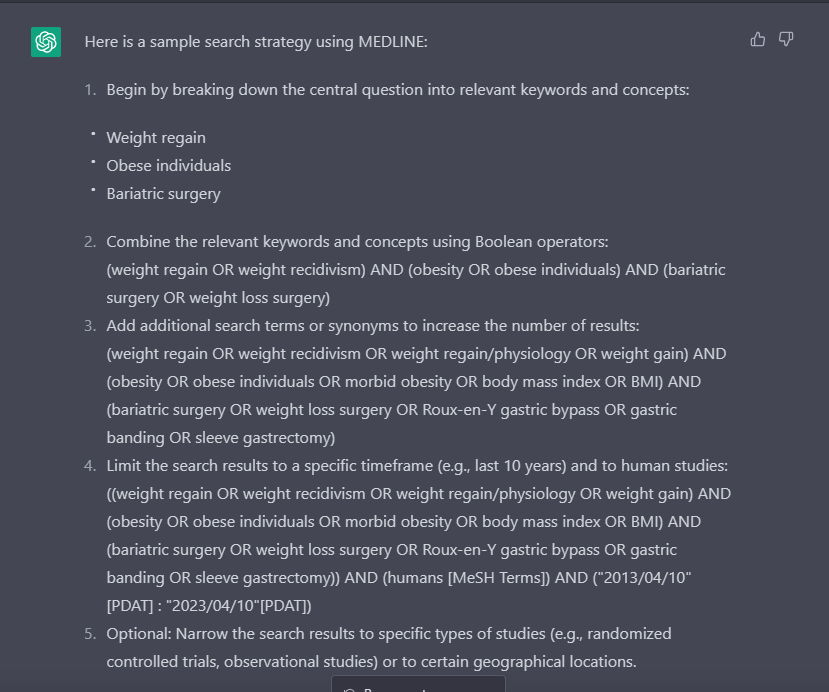

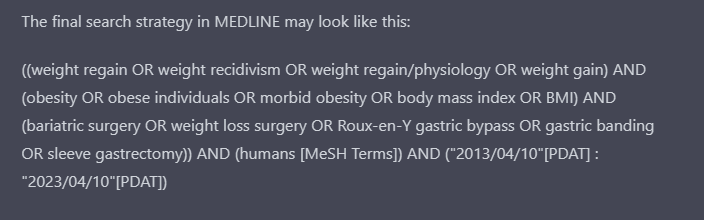
**Figure 1** - General guidance on building search strategies.


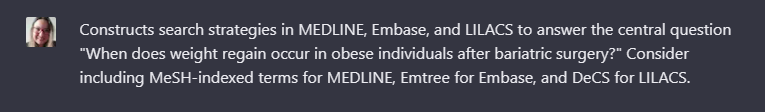


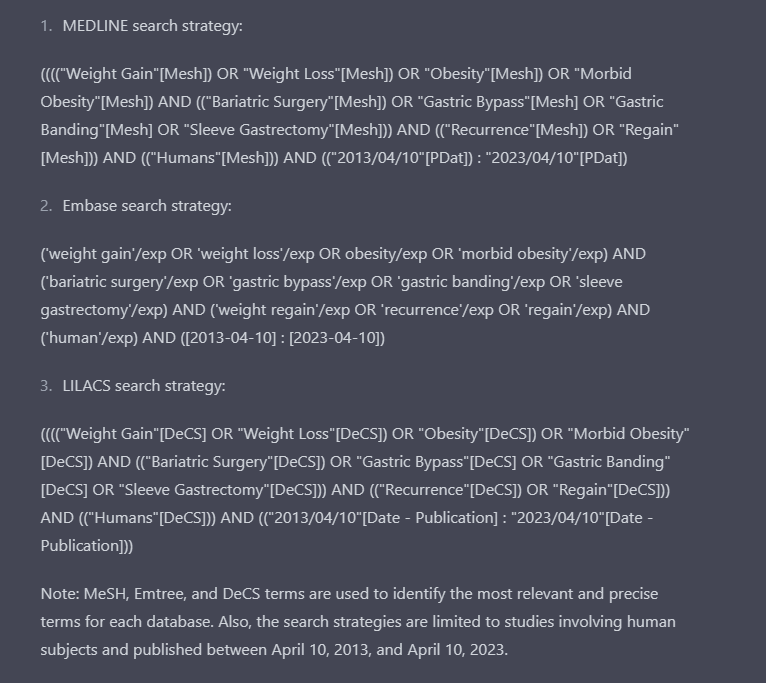
**Figure 2 -** Specific orientation for the construction of search strategies for information in electronic databases: MEDLINE, Embase.com, and LILACS.

| **MEDLINE** |
| --- |
| #1 “"Body-Weight Trajectory"[Mesh] OR "Body Weight Trajectory" OR "Body-Weight Trajectories" OR "Weight Trajectory" OR "Trajectories, Weight" OR "Trajectory, Weight" OR "Weight Trajectories" OR "Weight Change Trajectory" OR "Weight Change Trajectories" OR "Weight Gain Trajectory" OR "Trajectories, Weight Gain" OR "Trajectory, Weight Gain" OR "Weight Gain"[Mesh] OR "Gain, Weight" OR "Gains, Weight" OR "Weight Gains" OR "Weight Cycling" OR "Cycling, Weight" OR "weight regain" OR "weight recidivism" OR "relapse*" OR "recrudescence*" OR "weight failure"  #2  “Bariatric Surgery” [Mesh] OR “Surgeries, Bariatric” OR “Surgery, Bariatric” OR “Metabolic Surgery” OR “Metabolic Surgeries” OR “Surgeries, Metabolic” OR “Surgery, Metabolic” OR “Bariatric Surgical Procedures” OR “Bariatric Surgical Procedure” OR “Procedure, Bariatric Surgical” OR “Procedures, Bariatric Surgical” OR “Surgical Procedure, Bariatric” OR “Surgical Procedures, Bariatric” OR “Bariatric Surgeries” OR “Stomach Stapling” OR “Stapling, Stomach”  #3 Obesity [Mesh] OR “obesity” OR “obese”  #4  ((clinical[Title/Abstract] AND trial[Title/Abstract]) OR clinical trials as topic[MeSH Terms] OR clinical trial[Publication Type] OR random*[Title/Abstract] OR random allocation[MeSH Terms] OR therapeutic use[MeSH Subheading]) |
| **EMBASE.COM** |
| ('body weight change'/syn OR 'weight regain'/syn OR 'weight management'/syn OR ‘weight relapse’/syn) AND [embase]/lim  AND  ('Bariatric Surgery'/syn) AND [embase]/lim  AND  ('obesity'/syn OR 'obese patient'/syn) AND [embase]/lim  AND  'clinical trial'/de OR 'randomized controlled trial'/de OR 'randomization'/de OR 'single blind procedure'/de OR 'double blind procedure'/de OR 'crossover procedure'/de OR 'placebo'/de OR 'prospective study'/de OR ('randomi?ed controlled' NEXT/1 trial*) OR rct OR 'randomly allocated' OR 'allocated randomly' OR 'random allocation' OR (allocated NEAR/2 random) OR (single NEXT/1 blind*) OR (double NEXT/1 blind*) OR ((treble OR triple) NEAR/1 blind*) OR placebo* |

**Figure 3** - Manual Search Strategies.
